# Organotrialkoxysilane-Functionalized Prussian Blue Nanoparticles-Mediated Fluorescence Sensing of Arsenic(III)

**DOI:** 10.3390/nano11051145

**Published:** 2021-04-28

**Authors:** Prem. C. Pandey, Shubhangi Shukla, Roger J. Narayan

**Affiliations:** 1 Department of Chemistry, Indian Institute of Technology (BHU), Varanasi 221005, India; pcpandey.apc@iitbhu.ac.in (P.C.P.); shubhangi.rs.chy14@itbhu.ac.in (S.S.); 2 Joint Department of Biomedical Engineering, University of North Carolina, Chapel Hill, NC 27599, USA

**Keywords:** prussian blue nanoparticles, organotrialkoxysilane, silica beads, arsenite, arsenate, water decontamination

## Abstract

Prussian blue nanoparticles (PBN) exhibit selective fluorescence quenching behavior with heavy metal ions; in addition, they possess characteristic oxidant properties both for liquid–liquid and liquid–solid interface catalysis. Here, we propose to study the detection and efficient removal of toxic arsenic(III) species by materializing these dual functions of PBN. A sophisticated PBN-sensitized fluorometric switching system for dosage-dependent detection of As^3+^ along with PBN-integrated SiO_2_ platforms as a column adsorbent for biphasic oxidation and elimination of As^3+^ have been developed. Colloidal PBN were obtained by a facile two-step process involving chemical reduction in the presence of 2-(3,4-epoxycyclohexyl)ethyl trimethoxysilane (EETMSi) and cyclohexanone as reducing agents, while heterogeneous systems were formulated via EETMSi, which triggered in situ growth of PBN inside the three-dimensional framework of silica gel and silica nanoparticles (SiO_2_). PBN-induced quenching of the emission signal was recorded with an As^3+^ concentration (0.05–1.6 ppm)-dependent fluorometric titration system, owing to the potential excitation window of PBN (at 480–500 nm), which ultimately restricts the radiative energy transfer. The detection limit for this arrangement is estimated around 0.025 ppm. Furthermore, the mesoporous and macroporous PBN-integrated SiO_2_ arrangements might act as stationary phase in chromatographic studies to significantly remove As^3+^. Besides physisorption, significant electron exchange between Fe^3+^/Fe^2+^ lattice points and As^3+^ ions enable complete conversion to less toxic As^5+^ ions with the repeated influx of mobile phase. PBN-integrated SiO_2_ matrices were successfully restored after segregating the target ions. This study indicates that PBN and PBN-integrated SiO_2_ platforms may enable straightforward and low-cost removal of arsenic from contaminated water.

## 1. Introduction

Large numbers of people in Bangladesh and India are exposed to arsenic contamination in potable water. Metallurgical, agricultural, and industrial processes result in the discharge of arsenic into soil and water [1,2]. Long-term exposure to arsenic, even at low concentrations, can lead to oncological, immunological, neurological, and endocrine effects [3]. The World Health Organization recently set an arsenic limit of 10 μg/L for drinking water (Holm, 2002) [4]. Natural water predominantly contains the inorganic species arsenate [HAsO_4_^2^^−^, As(V)] and arsenite [AsO_2_^−^, As(III)]. Inorganic As(III) was noted to be more toxic (10 times), mobile, and water-soluble (4–10 times) than As(V) [5]. The conversion rate of As(III) (arsenite) to As(V) (arsenate) in oxygenated water is a slow process, which depends on certain specific conditions [6]. Consequently, there is an alarming need to develop novel methods for sensing and removal of arsenic from drinking water [7].

Prussian blue nanoparticles (PBN) contain metal in two different oxidation states, Fe^+3^ and Fe^+2^; these materials are known for their advanced peroxidase mimetic activity [8,9,10]. The charge transfer between the two iron species is responsible for the deep blue color of the complex [11]. Bi-metallic coordination compound PBN are a well-known inorganic material for electrocatalytic applications [12,13,14,15]. Several reports demonstrated the formation of mixed metal analogues, which involve straightforward replacement of the ferric/ferrous ion with another metal having a similar chemical state [16,17,18]. The properties of Prussian blue complex can be readily modified depending upon the nature of the constituent metal pair. Iron hexacyanoferrate synthesized via traditional synthetic routes (e.g., co-precipitation and electrosynthesis) do not exhibit appropriate processability for technical applications. We processed PBN from a single precursor involving the active role of the organotrialkoxysilane, which not only controlled the nucleation and solubility but also provided stability to the contents of reaction medium [19]. In addition, the PBN made from a single precursor were found to act as a light quenching material [20]; the photoactivity of the materials was examined using fluorescence imaging. Earlier studies show that organotrialkoxysilanes such as 3-aminopropyltrimethoxysilane (APTMS) allow the conversion of a single precursor, potassium hexacyanoferrate, to Prussian blue; this material was used for electrocatalytic detection of dopamine [21]. We further examined the use of another organotrialkoxysilane, 2-(3,4-epoxycyclohexyl) ethyltrimethoxysilane (EETMSi), in the presence of cyclohexanone for the controlled synthesis of PBN as a light quenching material.

Several methods, including iron oxide-coated sand, manganese greens, and iron ores, were previously described for arsenic removal [22]. Spectrophotometric and fluorometric methods have been previously studied to estimate the trace amounts of arsenite in water [23,24,25]. PBN, which include iron of two different oxidation states in a metal framework, may undergo specific interactions with As(III). Thus, we examined the fluorescence quenching ability of the PBN in the presence of arsenic(III). A novel result based on fluorescent sensing of arsenic was recorded, indicating the interaction between PBN and arsenic(III). PBN within a matrix were subsequently studied for use in arsenic removal.

Silica (SiO_2_) beads are a non-toxic and inexpensive matrix, which may be used as a template to synthesize PBN using organotrialkoxysilane. PBN were inserted into mesoporous SiO_2_; the PBN became embedded in the accessible SiO_2_ pores. The PBN@SiO_2_ was used for As(III) removal and its subsequent oxidation into arsenate through an interaction with the iron species in the material. This adsorption–oxidation process was demonstrated with PBN@SiO_2_ under different pH conditions to analyze the efficacy of the oxidant system. The high uptake efficiency of PBN@SiO_2_ (95%) indicated that this material is attractive for use in As(III) removal. XPS, ICP, and HPLC techniques were used to detect and quantify As(III) species. The PBN@SiO_2_ was separated easily through centrifugation; this recycled material also showed As(III) removal activity. The proposed As(III) removal process is more cost effective over those reported to date. The ability to recycle PBN@SiO_2_ adds to the economic viability of this process.

## 2. Experimental Section

### 2.1. Materials

Potassium ferricyanide was purchased from Merck India (Bengaluru, Karnataka, India). Silica beads (50 μm) and silica nanoparticles (200 nm) were purchased from Sigma-Aldrich (Bengaluru, Karnataka, India). Sodium arsenite was purchased from S D Fine-chem Limited (Mumbai, Maharashtra, India), and Azure-B was obtained from Sisco Research Laboratories Pvt. Ltd. (Mumbai, Maharashtra, India). 2-(3,4-epoxycyclohexyl)ethyltrimethoxysilane (EETMSi) and cyclohexanone were obtained from Sigma-Aldrich (Bengaluru, Karnataka, India). In addition, the remaining chemicals were of analytical grade and procured from commercial sources. The working solution of As(III) was freshly prepared with Milli-Q water using sodium arsenite (NaAsO_2_) and stored in a dark freezer. Milli-Q water was used throughout the experiment to avoid interference from contaminants.

### 2.2. Synthesis of PBN, PBN@SiO_2_ and PBN@MSNP Mediated through EETMSi

#### 2.2.1. EETMSi-Mediated Formation of PBN

The synthesis of PBN was accomplished using [2-(3,4-Epoxycyclohexyl)ethyl]trimethoxysilane(EETMSi) and cyclohexanone from the single precursor potassium ferricyanide via chemical reduction. The homogeneous colloidal sol of Prussian blue nanoparticles (PBN) was prepared by adding 20 μL of EETMSi (0.1 M) to 100 μL of potassium ferricyanide (0.03 M) under stirring conditions. Subsequently, 20 μL of cyclohexanone was added to the reaction mixture; this mixture was kept in an oven at 343 K for 8 h. The blue-colored colloidal suspension of PBN was characterized byX-ray diffraction (XRD), Transmission electron micrpscopy (TEM), etc.

#### 2.2.2. EETMSi-Mediated Formation of Prussian Blue Nanoparticles Modified Silica (PBN@SiO_2_)

Mesoporous silica was used to obtain PBN-confined mesoporous silica (PBN@SiO_2_). Typical synthesis involved a multistep procedure as follows: At first, 10 mg of mesoporous silica beads were suspended in 100 mL of EETMSi (1.2 M) aqueous solution under constant stirring conditions. After 3 h, un-adsorbed EETMSi was extracted with methanol, followed by centrifugation. 200 mL of potassium ferricyanide [K_3_Fe(CN)_6_] aqueous solution (0.03 M) was added to the alkoxysilane-modified SiO_2_ suspension under vigorous stirring conditions (800 rpm). Cyclohexanone was added to the alkoxysilane-modified K_3_Fe(CN)_6_@SiO_2_ suspension under vigorous stirring and left to stand in oven at 338 K overnight. The unreacted K_3_Fe(CN)_6_ and unabsorbed PBN were removed via washing (five times) with methanol/water (2:1) solvent. The residual material was collected after centrifugation; a drying step was subsequently performed.

#### 2.2.3. EETMSi-Mediated Formation of PBN@MSNPs

Mesoporous silica nanoparticles (MSNPs) were used to prepare Prussian blue nanoparticle-embedded mesoporous silica nanoparticles (PBN@MSNP). Ten milligrams of mesoporous silica nanoparticles (average particle size 200 nm and pore size 6 nm) were suspended in 100 mL of EETMSi (1.2 M) aqueous solution under stirring conditions. After 3 h, un-adsorbed EETMSi was removed with methanol, followed by centrifugation. Two hundred milliliters of potassium ferricyanide aqueous solution (0.03 M) were added to the alkoxysilane-modified MSNP suspension under vigorous stirring conditions (800 rpm). Cyclohexanone was added to the alkoxysilane-modified K_3_Fe(CN)_6_@MSNPs suspension under continuous stirring and left to stand in oven at 338 K overnight. The unreacted K_3_Fe(CN)_6_ and unabsorbed PBN were removed via washing (five times) with methanol/water (2:1) solvent. The residual material (PBN@MSNPs) was collected after centrifugation; a drying step was subsequently performed.

### 2.3. Materials Characterization

The particle size and morphology of as-synthesized PBN/PBN@SiO_2_ and PBN@MSNP were analyzed using high-resolution transmission electron microscopy (HRTEM) with 800 and 8100 instruments (Hitachi, Tokyo, Japan) at an acceleration voltage of 200 kV. The topographical properties of as-synthesized PBN over SiO_2_ were analyzed using a field emission scanning electron microscopy instrument (FEI (S.E.A.) Pte Ltd., Singapore). The elemental confirmation and mapping analyses were accomplished with an EDX attachment (Oxford Instruments plc, Abingdon, UK). A Rigaku X-ray diffractometer (Rikagu, Tokyo, Japan) with Cu Ka radiation (λ = l.5406 A^0^) was used to evaluate diffraction data. The XRD analysis was performed over the scan range of 10–90° for PBN. FTIR spectra were recorded on an ALFA-ATR Fourier transform infrared spectrometer (Bruker, Ettington, Germany). XPS analysis was performed using an ESCA/AES System (Surface Nano Analysis, GmbH, Berlin, Germany), which was equipped with an Al-Kα (1486.6 eV) X-ray source operating at a power of 385 W and a PHOBIOS 150 3D energy hemispherical analyzer with a delayline detector (SPECS Surface Nano Analysis GmbH, Berlin, Germany). The C-1s peak (284.5 eV) was used as an internal reference to calibrate the absolute binding energy. The quantitative detection of elements was performed through ICP techniques. Fluorescence analysis was performed using a 7100 spectrophotometer (Hitachi, Tokyo, Japan). Arsenic speciation was performed using high-performance liquid chromatography (HPLC) with a Shim-packed GIST C18 chromatography column encompassing a hydrophobic (non-polar) stationary phase (column length = 75 mm, inner diameter = 7.6 mm) for the determination of all species. Ammonium phosphate solution was used as an eluent for the entire HPLC experiment. The HPLC mobile phases of ammonium phosphate solution with pH 6.9 were prepared by mixing monobasic (NH_4_H_2_PO_4_) and dibasic ((NH_4_)_2_HPO_4_) salt solutions with an appropriate ratio.

### 2.4. Fluorometric Method

A fluorometric method was used for the determination of As(III) species. Fluorescein (Flo) was used as a probe molecule (λex = 480 nm, λem = 510 nm) for the estimation of As(III) species. The fluorescence experiment was performed under neutral pH (6.8) conditions using Milli-Q water. Different concentrations of As(III) standard solution (10 ppm to 320 ppm) were prepared by adding appropriate amounts of sodium arsenite to Milli-Q water. The result was obtained using the effective concentration of Flo, PBN, and As(III).

## 3. Results and Discussion

### 3.1. Organotrialkoxysilane-Mediated Synthesis of PBN Analogs

**Figure 1:**
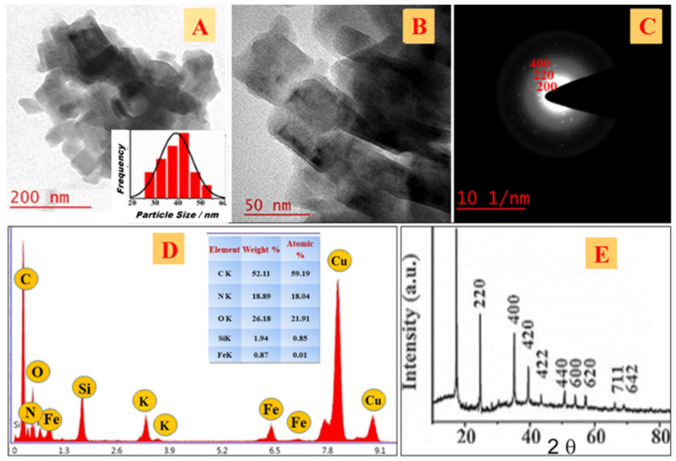
TEM images of PBN at different magnifications (**A**,**B**). Bar histogram displaying the particle size distribution curve of the nanoparticles (inset of Figure 1A). SAED pattern of as-synthesized particles (**C**), EDX profile with all the anticipated elements (**D**), XRD of EETMSi-functionalized PBN (**E**).

Organotrialkoxysilane with an amine functional group, APTMS, in the presence of cyclohexanone was previously used for the controlled conversion of a single precursor, K_3_[Fe(CN)_6_, into Prussian blue nanoparticles under ambient conditions [20]. Subsequently, 2-(3,4-epoxycyclohexyl) ethyltrimethoxysilane (EETMSi) was used to make PBN in the absence of cyclohexanone [9]. Although the process enabled efficient conversion of single precursor, K_3_[Fe(CN)_6_, into Prussian blue nanoparticles, the duration was substantially longer. Accordingly, we attempted to use cyclohexanone along with EETMSi to obtain PBN from a single precursor pathway. Indeed, the process enabled the rapid formation of PBN, as shown in Figure 1; additional details on this process are provided below.

#### 3.1.1. PBN as a Homogeneous Suspension

Slow decomposition under hydrothermal conditions via single precursor synthesis readily produced a blue-colored solution of PBN. The TEM micrographs in Figure 1A,B revealed well-dispersed nanocubes of PBN with an average diameter of 30 nm. The histogram (inset of Figure 1A) shows broad size distribution of crystalline nanoparticles, ranging between 27 and 53 nm. The average width of nanoparticles may be altered by modifying the EETMSi/Fe^3+^/cyclohexanone feed ratio and thermal conditions. Accordingly, we investigated the role of EETMSi in combination with a ketonic reducing agent. The EDX and TEM data provided information on the chemical composition and nanoparticle structure, respectively. Figure 1D shows the contributions to the EDX spectrum from the Fe Kα peak at 6.4–7.0 keV and 0.9 keV, the Cu peak at 7.8–9.0 keV, and the Si peak at 7.057 keV; Fe peak and the peaks for N, O, and C are also noted. The XRD spectrum shown in Figure 1E reveals nearly all the planes assigned to 2θ values as per JCPDS # 73-0687, 17.4° (200), 24.7° (220), 35.3° (400), 39.6° (420), and 43.7° (422), 50.0° (440), 53.9° (600), 57.2° (620), 66.1° (640), and 68.9° (642).

#### 3.1.2. PBN Confined in Mesoporous Silica (PBN@SiO_2_)

**Figure 2:**
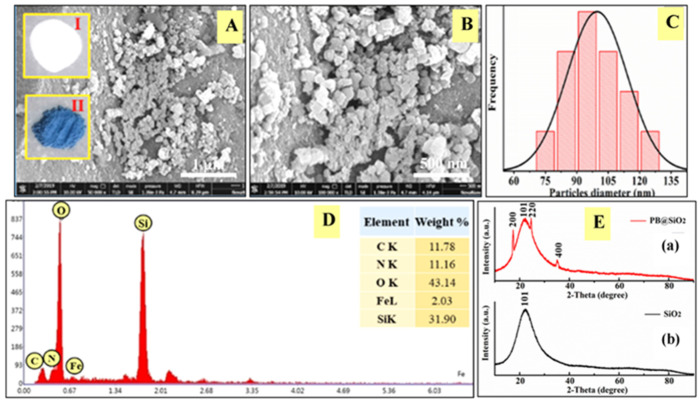
(**A**,**B**) HRSEM images of PBN@SiO_2_ at two different magnifications. Inset of (**A**) shows photographic images of mesoporous silica (I) and PBN-inserted mesoporous silica (II). (**C**) The particle size distribution of PBN within mesoporous silica. (**D**) The EDX spectrum of PBN-inserted mesoporous silica. (**E**) XRD spectra of SiO_2_ (**a**) and PBN@SiO_2_ (**b**).

We also attempted to insert PBN into mesoporous silica through the synergistic action of EETMSi and cyclohexanone; the product is represented as PBN@SiO_2_. Mesoporous silica with a particle size of 50 micrometers and a pore diameter of 6 nm was used for the synthetic insertion of PBN; these materials have use in column chromatography. SEM micrographs in Figure 2A–C show the topographical features of PBN@SiO_2_ and the narrow size distribution of the PBN in the SiO_2_ matrix. Figure 2D shows the EDX data for PBN@SiO_2_, which shows a silicon content of 31.9% elemental weight; the inset of Figure 2A shows photographic images of mesoporous SiO_2_ (I) and PBN@SiO_2_ (II). The XRD spectra of as-made PBN@SiO_2_ and mesoporous SiO_2_ are shown in Figure 2E(a–b). The results for as-made PBN@SiO_2_ and mesoporous SiO_2_ demonstrate a broad peak, which is assigned to the 101 plane of amorphous SiO_2_ (Figure 2E(a)); additional peaks for as-made PBN@SiO_2_ are assigned to 220, 220 and 400 lattice planes of crystalline PBN. After exposure to the EETMSi-mediated PBN-laden formulation, an alteration in the SiO_2_ pore size was detected via BET analysis (Table 1).

#### 3.1.3. PBN-Doped Mesoporous Silica Nanoparticles (PBN@MSNP)

**Figure 3:**
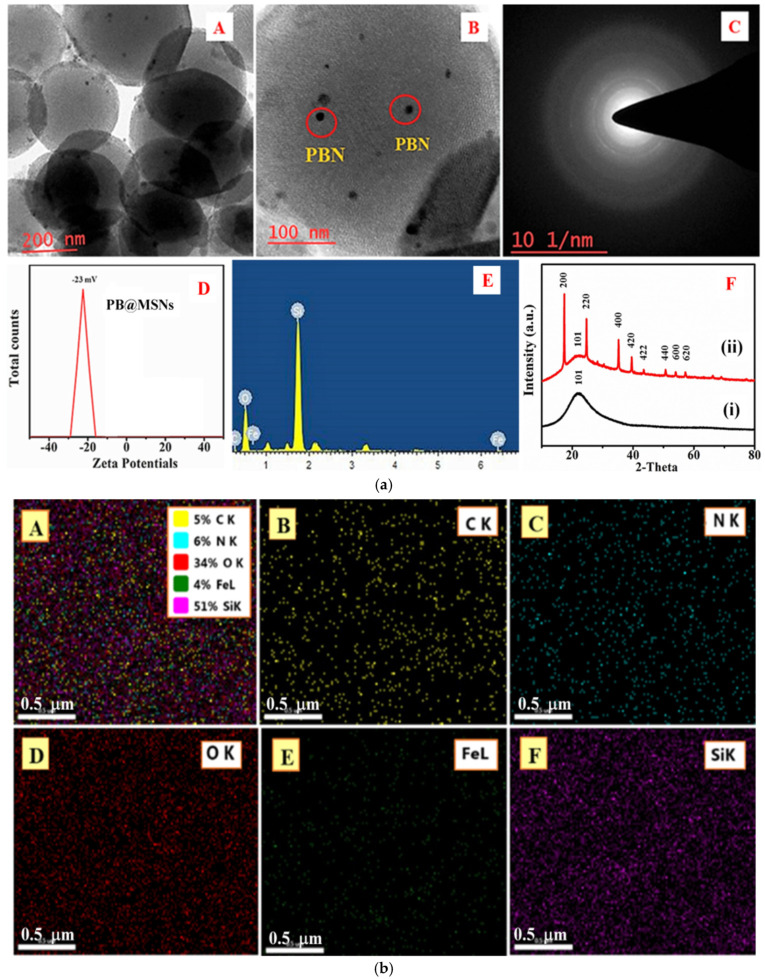
(**a**) (**A**) HRTEM image of organotrialkoxysilane-functionalized Prussian blue nanoparticles (PBN@MSN), (**B**) micrograph showing the magnified view of bulk-confined PBN (encircled in red) in mesoporous silica, (**C**) SAED pattern of the corresponding hybrid nanoparticle assembly (PBN@MSN), (**D**) stability profile of PBN@MSN in terms of zeta potential measurement, and (**E**) EDX spectrum of PBN@MSNP. (**F**) XRD profile for MSNPs (i) and as-synthesized PBN@MSNPs (ii). (**b**) (**A**) Mapping analysis of organotrialkoxysilane-functionalized Prussian blue nanoparticles with elemental composition (**B**) carbon, (**C**) nitrogen, (**D**) oxygen (**E**) iron, and (**F**) silicon.

We also undertook the synthetic incorporation of PBN within mesoporous silica nanoparticles. Silica nanoparticles (MSNP) with an average particle size of 200 nm and a pore size of 6 nm were used for this purpose. The porous nanocomposite was obtained primarily in two steps: (a) surface functionalization of the matrix by EETMSi, followed by (b) the uniform distribution of metal precursor throughout the network and subsequent reduction to form nanoscale particles. The in situ growth of PBN was pH controlled. The soluble Fe^3+^ species easily adhered to the pore channels in the presence of capping agent EETMSi. The HRTEM micrograph of bare MSNPs (Figure 3a(A)) shows a porous skeleton of spherical morphology. Figure 3a(B) shows PBN inside the mesoporous silica nanoparticles (encircled in red) [26]. The selected area electron diffraction (SAED) pattern of the corresponding hybrid nanoparticle assembly (PBN@MSNP) is shown in Figure 3a(C). The zeta potential value was obtained from dynamic light scattering (DLS) data to understand the solution stability of particles. As shown in Figure 3a(D), the value of zeta potential is nearly −23 mV (i.e., towards the negative side); hence, the PBNPs are also negatively charged.

The EDX spectrum of PBN@MSNPs is shown in Figure 3a(E). The EDX mapping of the organotrialkoxysilane-functionalized PBN@MSNPs with the elemental composition of (B) carbon, (C) nitrogen, (D) oxygen (E) iron, and (F) silicon is shown in Figure 3b. The crystallographic data for as-prepared PBN@MSNPs and blank MSNPs are shown in Figure 3a(F). The peaks indexed at 2θ values of 17.6° (200), 24.3° (220), and 37.8° (400) indicated the successful insertion of crystalline PBN within the SiO_2_ matrix; per JCPDS # 73-0687, 17.4° (200), 24.7° (220), 35.3° (400), 39.6° (420), and 43.7° (422), 50.0° (440), 53.9° (600), 57.2° (620), 66.1° (640), 68.9° (642), and 77.2° (820) can be indexed as the PB cubic space group Fm3m.

### 3.2. FTIR Analysis of PBN@SiO_2_

**Figure 4:**
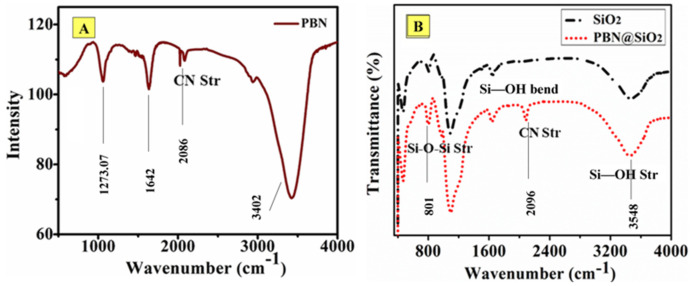
(**A**) FTIR spectrum of as-synthesized PBN; (**B**) FTIR spectra of (black line) SiO_2_ and (red line) as-synthesized PBN@SiO_2_.

The peak at 2086 cm^−1^ in the FTIR spectrum (Figure 4A of PBN may be attributed to the CN stretching mode of the Fe(II)-C-N-(III)Fe moiety in PBN. The broad bands at 3402 cm^−1^ and 1642 cm^−1^ in the spectrum correspond to OH-stretching and H_2_O bending mode of the interstitial water molecule, respectively, within the PBN lattice. The strong band near 2885–2990 cm^−1^ corresponds to the C-H stretching vibration of sp^2^-hybridized carbon in cyclohexanone.

The broad bands centered at 3548 cm^−1^ and at 1632 cm^−1^ are assigned to the stretching and bending vibrations of silanol groups (Si–OH), respectively, in the silica beads [27]. The bands at 1093 cm^−1^ and 801 cm^−1^ in the spectrum are associated with the anti-symmetric and symmetric stretching modes (Si–O–Si) of SiO_4_ units. The prominent peak at 2096 cm^−1^ (Figure 4B is attributed to the stretching mode of Fe(II)-CN-(III)-Fe moiety in PBN [28] and indicates the successful formation of nanoparticles over SiO_2_.

### 3.3. Fluorometric Study

#### 3.3.1. Effect of the Addition of As(III) on the Fluorescent Intensity of Fluorescein

**Figure 5:**
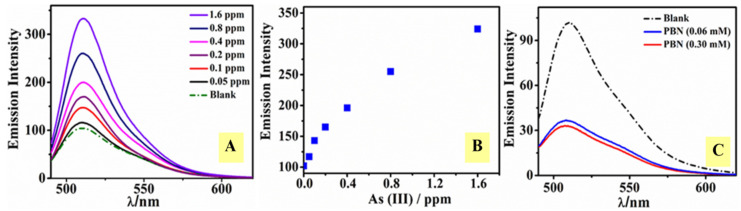
Fluorescence emission spectra of Flo (blank) in the presence of different As(III) concentrations from 0.05 ppm to 1.6 ppm (**A**). Plot of fluorescence intensity of Flo (0.2 mM) after exposure to a variable concentration (0.05–1.6 ppm) of As(III) (**B**). Effect of PBN addition (0.06 mM, 0.3 mM) over Flo (0.2 mM) fluorescence (**C**).

Since PBN have already been established as light quenching material, the PBN-mediated fluorescence quenching of fluorescein was evaluated. The impact of As(III) on fluorophore activity was studied via adding a different concentration of As(III) solution to a fixed Flo concentration. Subsequently, 0.01 mL of As(III) (10–320 ppm) and 10 μL of Flo solution (0.2 mM) were transferred into 2 mL of Milli-Q water and allowed to stand for 2 min at room temperature prior to fluorescence analysis. The fluorescence intensity of Flo was found to be enhanced as the function of As(III) (Figure 5A,B). At a lower As(III) concentration, a less pronounced enhancement phenomenon was observed. This result revealed that the extent of the interaction between Flo molecule and As(III) occurred to a higher extent at a higher As(III) concentration (up to 1.5 fold). The emission intensity was found to enhanced three-fold when the concentration of As(III) was elevated from 0.05 ppm to 2 ppm (effective concentration).

#### 3.3.2. Interaction of Fluorophore with PBN

PBN were employed to observe the effect of the nano-sized particles over Flo. A constant amount of PBN nanosol (0.3 mM) was added to a known concentration of Flo (0.2 mM); the mixture was allowed to stand at room temperature for 2 min. The results revealed that PBN quenched the fluorescence property of Flo as shown in Figure 5C. Furthermore, the effect of PBN concentration over the emission intensity of Flo was investigated. Mixtures containing various concentrations of PBN with Flo were used to understand the interaction of nanoparticles with the fluorophore. The mixtures (i) PBN (0.06 mM) with Flo (0.2 mM) and (ii) PBN (0.3 mM) with Flo (0.2 mM) were evaluated. It was shown that EETMSi functionalized PBN acted as a quencher for Flo since the intensity of the fluorophore was found to diminish in the presence of PBN (Figure 5C). On increasing the concentration of PBN from 0.06 mM to 0.3 mM, only a small reduction in the emission intensity was observed (Figure 5C).

#### 3.3.3. Effect of As(III)/PBN System over Flo Intensity

**Figure 6:**
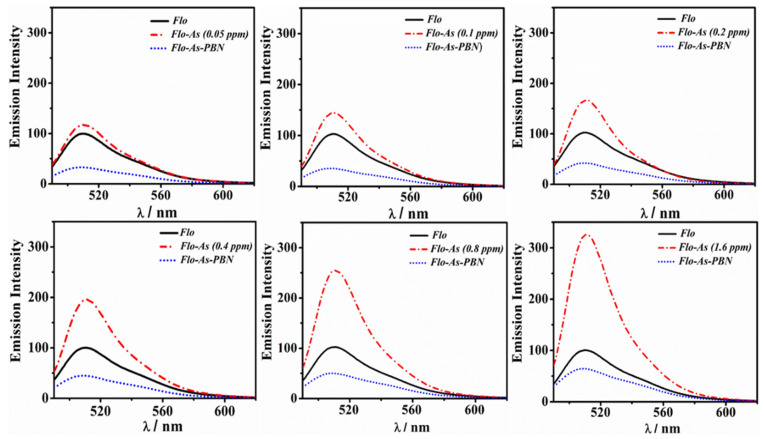
Study of the impact of PBN addition (10^−l^) over the emission intensity of Flo-As(III) system by varying the As(III) solution (0.05–1.6 ppm) and keeping a constant concentration of Flo (0.2 mM) and PBN (0.3 mM).

**Figure 7:**
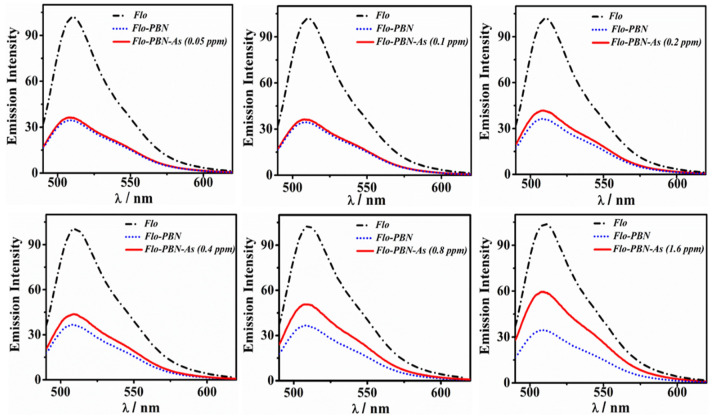
Study of the influence of As(III) addition over the emission spectra of the quenched Flo-PBN system by adding different concentrations of As(III) solution (0.05–1.6 ppm) and keeping a constant concentration of Flo (0.2 mM) and PBN (0.3 mM).

**Figure 8:**
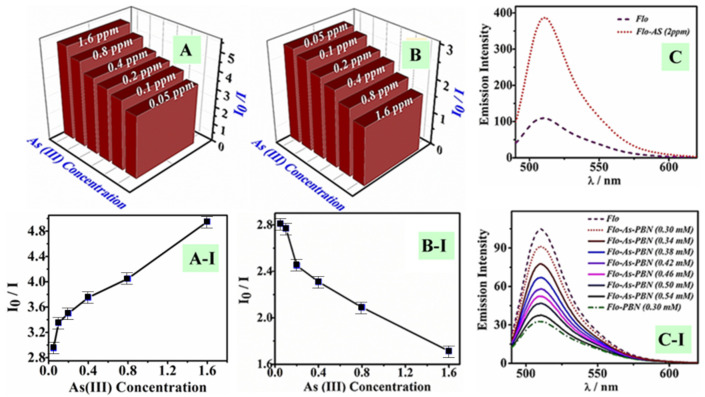
Bar diagram displaying fluorescence quenching (FQ) with respect to the variable concentration (effective concentration) of As(III) for the PBN@Flo-As(III) system (**A**) and the As(III)@PBN-Flo system (**B**). Plots of fluorescence intensity of the Flo-PBN system with respect to a variable content of As(III), showing concentration-dependent fluorescence quenching in both circumstances (**A-I**,**B-I**), with error bars representing the standard deviation. Emission spectra displaying the effect of adding 2 ppm of As(III) to the emission intensity of Flo (**C**). The required amount of PBN for complete quenching by adding various amounts of PBN to the Flo-As(III) system (**C-I**) and Flo.

To understand the active role of PBN over As(III) interaction, we performed two experiments. In the first experiment, different concentrations of As(III) varying from 0.05 ppm to 1.6 ppm (effective concentration) were added to the fixed content of Flo (0.3 mM); the Flo-As(III) system was then exposed to a constant amount of PBN (0.3 mM) (as shown in Figure 6). In the second experiment, PBN (0.3 mM) were initially added to the Flo solution (0.2 mM); a variable concentration of As(III) between 0.05 ppm and 1.6 ppm (effective concentration) was then added to the PBN-Flo system (Figure 7). The substantial fluorescence quenching of the Flo-As(III) system in the presence of PBN was calculated using the relation F_0_/F, where F_0_ and F denote the fluorescent intensity of the Flo-As(III) system in the absence and in the presence of PBN, respectively (Figure 8A). Similarly, the substantial fluorescence quenching of the Flo system in the presence of PBN was calculated using the relation F_0_/F, where F_0_ denotes the fluorescence intensity of the Flo system in the absence of PBN and As(III), and F denotes the fluorescence intensity of Flo in the presence of PBN and As(III) (Figure 8B).

It was observed that As(III) interacted predominately with the available quantity of the PBN moiety; the residual available As(III) was associated with the rise in emission intensity after interacting with Flo (Figure 8C-I). The subsequent addition of PBN achieved maximum quenching after interacting with available As(III), as displayed in Figure 8C-I. This result indicates that 0.54 mM PBN was sufficient to obtain complete interaction with 2 ppm arsenite. A similar concentration of 2 ppm of As(III) was added to the Flo-PBN system, which contained both PBN and Flo. The results (Figure 8A-I and Figure 8B-I) showed a decrease in fluorescent intensity of Flo-As(III) and Flo as a function of PBN; PBN altered the fluorescence influencing properties of As(III). It is surmised that Prussian blue interacted with As(III) more efficiently than the Flo-As system throughout the fluorescence process. A separate experiment was performed to discover the PBN loading for complete removal of As(III) from a concentration of 2 ppm (effective concentration). For this study, primary emission spectra of Flo-As(III) were recorded while adding As(III) aqueous solution (2 ppm) to the blank solution containing Flo (0.2 Mm) only (Figure 8C). A similar concentration of 2 ppm of As(III) was added to the Flo-PBN system, which contained both PBN.

### 3.4. As(III) Decontamination from Aqueous Solution Using PBN@SiO_2_

**Figure 9:**
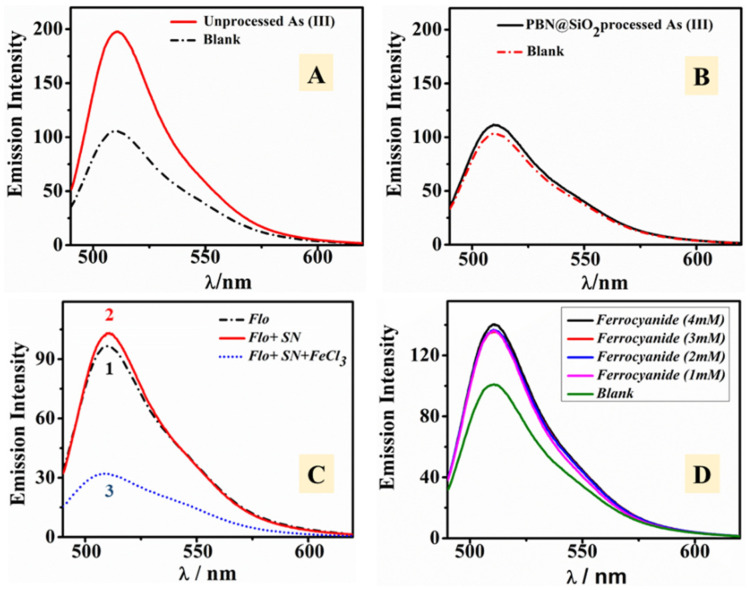
Fluorescence emission spectra of Flo with unprocessed (**A**) and PBN@SiO_2_ processed (**B**) As(III) solution. Identification of ferrocyanide species in supernatant on the addition of ferric chloride via fluorescence quenching (**C**). Study of the effect of ferrocyanide species (1–4 mm) over Flo emission spectra (**D**).

The heterogeneous PBN@SiO_2_ system was studied in order to understand the dynamic interaction occurring between As(III) and PBN. Accordingly, inexpensive and non-reactive silica beads were used for the modulation of active PBN in the formulation of the heterogeneous matrix. Heterogeneous methods are considered to play an influential role in catalysis due to their straightforward separation and large-scale applicability. For As(III) decontamination, the as-synthesized PBN@SiO_2_ (0.05 g) was successfully packed in a column of 10 mm diameter. The standard As(III) solution (10 ppm) was prepared via adding an appropriate amount of sodium arsenite salt in Milli-Q water; 10 mL of the solution was passed through the PBN@SiO_2_ enclosed column. The fluorescence analysis of separated supernatant (PBN@SiO_2_ processed) was performed using Flo (0.2 mM) under similar conditions. In this study, 10 μL of as-eluted supernatants (PBN@SiO_2_ processed and unprocessed As(III) solution) were added separately with Flo and left to stand at room temperature for 2 min. Their emission spectra were recorded to understand PBN@SiO_2_ interactions with As(III). Unprocessed As(III) solution was observed to enhance the emission intensity of Flo many-fold (Figure 9A) as compared to the PBN@SiO_2_ processed As(III) solution (Figure 9B).

PBN@SiO_2_ was shown to significantly remove the As(III) from the contaminated solution. The ICP analysis of PBN@SiO_2_ processed As(III) aqueous solution was performed to quantify the arsenic concentration in the solution. The result showed 0.0018 ppm arsenic (As) content for the PBN@SiO_2_ processed As(III) solution. In addition, 0.13 ppm Fe content was also detected in the processed As(III) solution. The ICP analysis indicated that some of the iron species of PBN ([Fe^III^[Fe^II^(CN)_6_]) leached out with the eluent during interaction with the As(III) species. To investigate the presence of active iron species in the eluent, we studied the addition of ferrous sulfate with active ferrous species (Fe^+2^) to the colorless supernatant eluent. During this process, we added the ferric chloride-containing active ferric species to the colorless supernatant eluent. We observed that colorless supernatant changed immediately to an intense blue color (resembling the Prussian blue color) when ferric chloride was added. However, no such changes were observed when ferrous sulfate (containing Fe^+2^) was added.

We performed a fluorometric experiment in which supernatant (SN) was employed to observe its modulation of the Flo fluorescence properties. Fluorescent emission spectra were recorded after adding Flo (10 μL) to 10 μL of PBN@SiO_2_ processed supernatant (SN). A small change in intensity (I_o_ = 102.23) was observed with respect to the Flo (I_o_ = 98.74) as seen in Figure 9C (1 and 2). A study that involved adding ferric chloride to the Flo-supernatant (Flo-SN) mixture showed that the Flo fluorescence property was quenched (I = 31.73) when compared to Flo (I = 98.74), as shown in Figure 9C (3). Supernatant-containing ferrocyanide species had an instant interaction with the added ferric chloride, which instantly converted into PBN.

To analyze the role of the residual ferrocyanide species in the supernatant over the emission spectra, a fluorescence experiment centered on the ferrocyanide concentration was conducted. We prepared and added different amounts (1 mM to 4 mM) of ferrocyanide solution to a constant amount of Flo (0.2 mM) to analyze the influence of the solution over the Flo emission intensity. Ferrocyanide acted as a weak enhancer (Figure 9D). These results indicate that the As(III) was supposed to undergo oxidation into arsenate in the presence of PBN. The iron species in the PBN undergo reduction into Fe^+2^ throughout the As(III) removal process. On the addition of active ferric species to the supernatant, an immediate reaction leads to the formation of PBN after the interaction with residual ferrous species. The collected PBN@SiO_2_ was characterized with XPS to observe the significance of arsenic treatment over the PBN@SiO_2_ phase (as discussed in a subsequent section). Moreover, the resultant eluent was collected into separate vials and underwent HPLC analysis for the detection of arsenic species.

### 3.5. HPLC Results on PBN@SiO_2_ Treated Arsenic(III)

**Figure 10:**
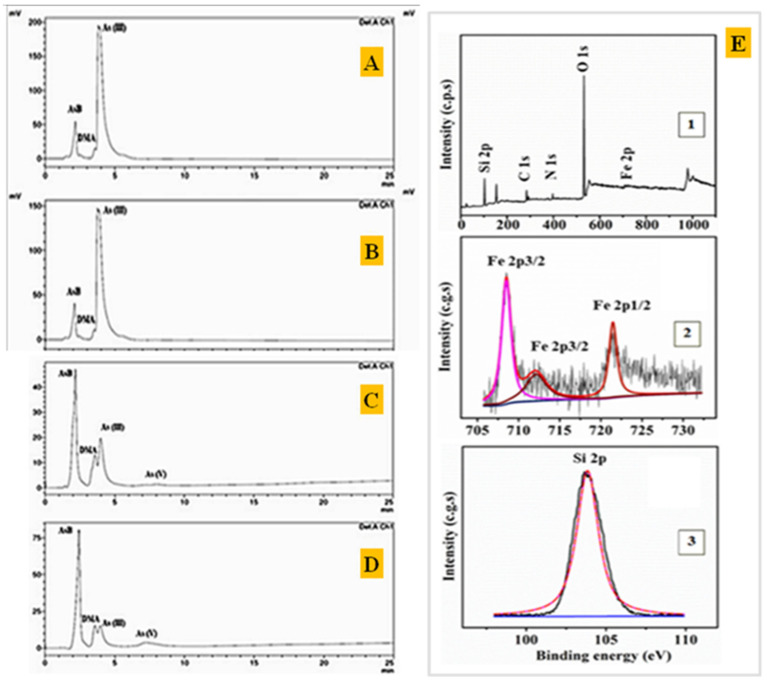
Ion chromatogram obtained during HPLC speciation of species present in the sample. (**A**) Standard As(III) (5 ppm) solution at pH = 6.5). (**B**) Standard As(III) sample (5 ppm) after PBN@SiO_2_ treatment in acidic medium (pH = 2). (**C**) As(III) standard solution (5 ppm) after PBN@SiO_2_ treatment in neutral medium (pH = 6.5). (**D**) As(III) standard sample (5 ppm) after PBN@SiO_2_ treatment in alkaline medium (pH = 8.5). (**E**) XPS analysis of PBN@SiO_2_. (**1**) A complete survey scan with all recognized species. (**2**) Fe^2+^ and Fe^3+^ species XPS peak in PBN@SiO_2_. (**3**) Identified Si(IV) chemical states in SiO_2_.

All the separated species were noted in the ion-chromatogram at their respective retention time such as arsenobetaine (AsB) at 2.17/2.42/2.55 min, dimethylarsinic acid (DMA) at 3.57 min, As(III) at 3.8/3.9 min, and As(V) at 7.7 min. The chromatogram shown in Figure 10A–D was obtained as the result of HPLC separation of the arsenic species after treatment with PBN@SiO_2_ at different pH values (2.2–8.5). HPLC analysis illustrates that the removal efficiency of As(III) ((Figure 10A) by PBN@SiO_2_ increased from 33.52% (Figure 10B) to 59.90% (Figure 10C) with a pH increase from 2 to 6.5; this improved to 95.13% (Figure 10D) under a mild alkaline condition (pH-8.5). The ion chromatogram results also showed an insignificant peak at a retention time of 7.35 min ((Area% = 1.4) at pH = 6.5 and (Area% = 9.09) at pH = 8.5), which was associated with leaching of As(V) in an aqueous solution during the oxidation–adsorption process. All of the arsenic species (As(III), DMA, AsB) identified at various retention times along with their relative concentration in a HPLC environment are shown in Table 2.

AsB, which frequently existed in the zwitterionic form due to the interaction between the positively charged arsenic and the negatively charged carboxylic group, starts to migrate immediately after interacting with the hydrophobic C18 Shim-pack column. However, As(III) is a neutral species (pKa = 9.2) up to a pH of 8, which eluents slowly with the solvent front. Consequently, negatively charged DMA and As(V) species feasibly eluent by a variety of interactions (e.g., H bonding and ion-exchange) along with hydrophobic effects. The obtained result was acquired after a total run time of 25 min and repeated twice to minimize the experimental error.

### 3.6. XPS Analysis of PBN@SiO_2_

XPS survey scans indicated the presence of Si, O, Fe, and C in blank PBN@SiO_2_ and As, Si, O, Fe, and C in As(III)-PBN@SiO_2_. The peaks were assigned as follows: Fe 2p_3/2_—708 eV; Fe 2p_3/2_—713 eV; Fe 2p_1/2_—722 eV; As(III) 3d—44.2 eV and As(IV) 3d—47 eV, respectively. The peak position of the Si 2p spectrum corresponds to a binding energy of 103.63 eV and shows the characteristics of Si(IV) in a SiO_2_-type compound [29].

#### 3.6.1. Fe(II) and Fe(III) Identification in PBN@SiO_2_

After peak fitting, the spectrum can be de-convolved into three peaks. Figure 10E shows the XPS peaks centered on binding energies of 721.27 and 708.34 eV for Fe 2p_1/2_ and Fe 2p_3/2_, respectively; these features are characteristic of the Fe^+2^ moiety in Prussian blue. In addition, a spectrum shows a peak at a binding energy of 712.12 eV, which corresponds to Fe^+3^ species. The position of these peaks is in good agreement with the results in the literature for the characteristic Fe^+3^ and Fe^+2^ components of Prussian blue compounds [30].

#### 3.6.2. As(III)-PBN@SiO_2_ and As(V)-PBN@SiO_2_

**Figure 11:**
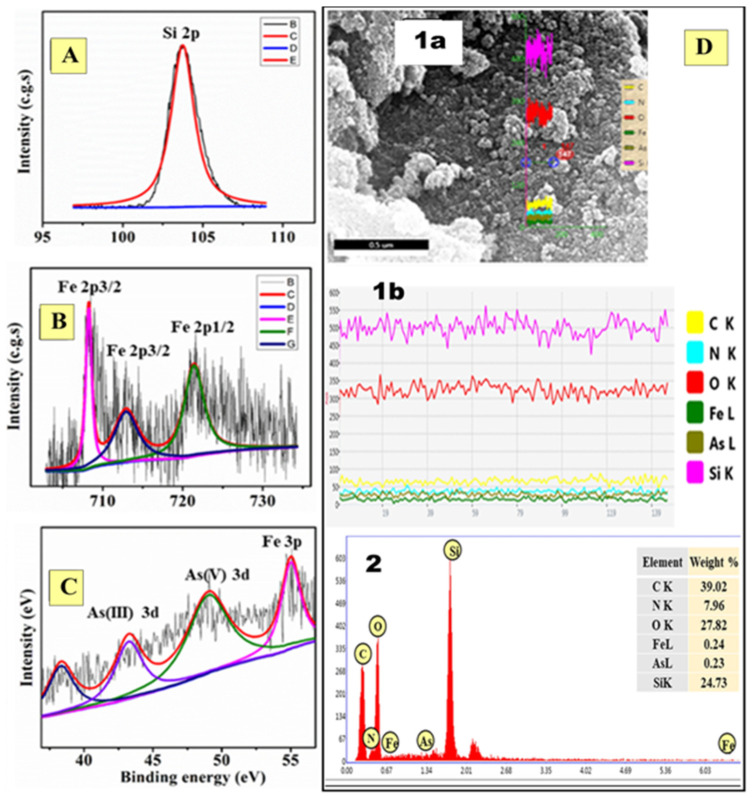
X-ray photoelectron spectrum of As-PBN@SiO_2_ after arsenic exposure. (**A**) Identified Si(IV) chemical states in SiO_2_. (**B**) Fe^2+^ and Fe^3+^ species XPS peak in PBN@SiO_2_. (**C**) Arsenic species (As(V) and As(III)) at PBN@SiO_2_. (**D**) (**1a**,**1b**) EDX line scan measurement comprised of an inner SiO_2_ and an outer ferric hexacyanoferrate with As(III) enrichment over the PBN@SiO_2_ surface. (**2**) EDX analysis shows all of the anticipated elements.

The cation As(III) and the oxidized species As(V) detected on the PBN@SiO_2_ substrate with XPS after a decontamination process are shown in Figure 11. The binding energy values (in eV) for O (1s), Si (2p), Fe (2p), and N (1s) in PBN@SiO_2_ and As-PBN@SiO_2_ are listed in Table 3. The XPS survey scan as shown in Figure 11D shows peaks at a binding energy of 49.03 eV, which are associated with the presence of As(V) and indicate the successful sorption of As(V) by PBN@SiO_2_ [31]. The other peak located at 43.23 eV is associated with the adsorption of As(III) over SiO_2_ prior to the oxidation process [32]. However, the peak positions observed for the Fe^+3^ and Fe^+2^ core level (2p) spectra of PBN@SiO_2_ are shifted slightly to a lower binding energy relative to the unreacted and unabsorbed PBN@SiO_2_ species. This shift in the peaks for Fe^2+^ 2p_3/2_ (binding energy of 708.19 eV) and Fe^2+^ 2p_1/2_ (binding energy of 721.42 eV) may be attributed to arsenic adsorption [33]. The shift in the peak position (with a reduction in intensity) of Fe^+3^ 2p_3/2_ (binding energy of 712.86 eV) relative to pure PBN@SiO_2_ suggests the reduction of the material during arsenic oxidation [34]. The position of the characteristic peak of Fe^+2^ (binding energy of 55.04 eV, 3p) remained unchanged throughout the As(III) oxidation and adsorption process [31]. A peak emerged at a binding energy of 398.99 eV, which was attributed to the presence of nitrogen in the environment. Alterations in the peak position of PBN@SiO_2_ relative to that of As-PBN@SiO_2_ were associated with chemical adsorption by PBN@SiO_2_ of arsenic species.

### 3.7. Effect of pH on Arsenic Removal

The results in Figure 10 illustrate the effects of pH on the removal of As(III) using PBN@SiO_2_. As can be observed, As(III) removal was dependent on pH; the greatest removal efficiency occurred under moderate pH (pH = 6–9) and was found to diminish at highly acidic pH (pH < 3). As reported earlier, surfaces of silica beads were positively charged in highly acidic conditions and acquired a negative charge in the pH range of 3–10 [35]. Subsequently, moderate pH was found to be favorable for a sorbent surface since decreased protonation is supposed to enlarge the attraction force between the negatively charged PBN@SiO_2_ surface and the positively charged As(III) cationic species. This result is similar to earlier findings by Gupta et al. who reported a significant increase in As(III) adsorption onto iron oxide-coated quartz sand with an increase in pH from 4.5 to 7.5 [36]. At a highly acidic pH (<3), repulsion occurred between the positively charged adsorbent sites and the adsorbate species (As^+3^), which prevented the adsorption and arsenic oxidation processes. No substantial rise in As(III) removal efficiency was observed with an elevation in pH.

### 3.8. Analysis of PBN@SiO_2_ Surface through SEM-EDX after As(III) Remediation

After the As(III) removal process, the PBN@SiO_2_ surface was analyzed using SEM. The result showed the change in morphology of PBN (cubic to spherical) after arsenic interaction (Figure 11E). The EDX results suggest that the material is comprised of an inner SiO_2_ chemistry and an outer ferric hexacyanoferrate (Fe^+3^[Fe^+2^(CN)_6_]) chemistry, with some As(III) enrichment over the PBN@SiO_2_ surface. The presence of the anticipated elements was confirmed through EDX analysis.

### 3.9. Recyclability and Proposed Mechanism

It has been well established that Prussian blue is comprised of Fe metal in Fe^+2^ (low spin) and Fe^+3^ (high spin) states, which are linked via CN bridges. Prussian blue can undergo reduction to what is known as Prussian white (Fe^II^–C≡N–Fe^II^) or oxidation to what is known as Prussian yellow (Fe^III^–C≡N–Fe^III^) [37,38]. Reduction of Prussian blue to Prussian white on the surface of silica gel was found to facilitate the As(III) oxidation to As(V) and their subsequent removal. Conversion of Fe^3+^ to Fe^+2^ was shown during the decontamination in PBN and validate the altered chemical environment due to arsenic interactions. The addition of ferric chloride to the white-blue colored arsenite-treated PBN@SiO_2_ residue instantly generated a blue color; this phenomenon is attributed to the conversion of hexacyanoferrate $\left[{\mathrm{Fe}}^{\mathrm{II}}{\left(\mathrm{CN}\right)}_{6}\right]$ species of $K_{2}[{\mathrm{Fe}}^{\mathrm{II}}{\mathrm{Fe}}^{\mathrm{II}}{\left(\mathrm{CN}\right)}_{6}]$ into Prussian blue $\left(K[{\mathrm{Fe}}^{\mathrm{III}}{\mathrm{Fe}}^{\mathrm{II}}{\left(\mathrm{CN}\right)}_{6}\right])$ through the interaction with ferric species (ferric chloride). The reaction during Prussian blue synthesis has been shown as:

$$4K_{2}[{\mathrm{Fe}}^{\mathrm{II}}{\mathrm{Fe}}^{\mathrm{II}}{\left(\mathrm{CN}\right)}_{6}]+4\mathrm{Fe}{Cl}_{3}4\left(K[{\mathrm{Fe}}^{\mathrm{III}}{\mathrm{Fe}}^{\mathrm{II}}{\left(\mathrm{CN}\right)}_{6}\right])+4\mathrm{KCl}$$

The variation of surface charge of SiO_2_ with a change in pH was found to be the fundamental framework for PBN activity over the course of arsenic removal.

### 3.10. Mechanism of PBN Based Fluorescence Sensing of As(III)

The findings as shown in Figure 4, Figure 5 and Figure 6 revealed an analyte-dependent intervalence transition in iron hexacyanoferrate [Fe^III^_4_[Fe^II^(CN)_6_]_3_] between Fe^2+^ and Fe^3+^ as shown below:

Fe^2+^ + Fe^3+^ + light energy → Fe^3+^ + Fe^2+^

The intervalence transition may be evaluated based on changes to the absorption spectrum. The fluorescein–PBN interaction is associated with fluorescence resonance energy transfer as recently described [39,40]; this material is capable of quenching the emitted fluorescence of fluorescein. When PBN undergo interaction with As(III), there is a conversion of PBN into Prussian white nanoparticles, followed by a conversion of As(III) to As(V); thus, the quenching ability is lost. The Prussian white nanoparticles can further be converted into PBN after treating the same with acid as discussed above. This scheme provides an effective and inexpensive method for PBN-mediated removal of As(III) udermvisible light.

### 3.11. Characterization of Recyclable PBN@SiO_2_

**Figure 12:**
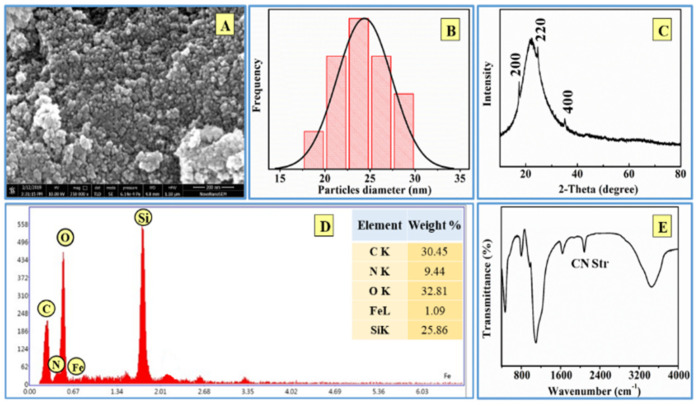
Analysis of recycled PBN@SiO_2_ following acid treatment. (**A**) An alteration in nanoparticle morphology as identified through SEM imaging, (**B**) A decrease in particle size (26 nm) as calculated from the displayed histogram, (**C**) Minor shift in peak position with similar planes as identified through XRD analysis, (**D**) Spectrum with the entire anticipated element as detected by EDX, (**E**) Shift in CN stretching peak observed using FTIR.

After arsenic elimination, the recycled PBN@SiO_2_ was investigated with XRD, SEM, and FTIR to understand the effect of the recycling process on PBN@SiO_2_ morphology, size, and crystallinity. An SEM image of the recycled PBN@SiO_2_ is shown in Figure 12. More spherical-shaped than cube-shaped particles were observed; the aggregation of PBN with no precise shape was also observed. XRD analysis demonstrated a shifting inward of the peak position (θ) as compared with the unused PBN@SiO_2_. The presence of anticipated elements was identified via EDX analysis. In addition, characteristic CN stretching in PBN was noted; this feature was noted at a considerably lower wavenumber (2054 cm^−1^), which is attributed to particle aggregation [39]. The PBN characteristics before and after the recycling process as obtained from XRD, SEM, and FTIR analysis are listed in Table 4.

## 4. Conclusions

PBN are a light-sensitive material that is processed through functional alkoxysilane- and cyclohexanone-mediated conversion of a single precursor, potassium hexacyanoferrate. The synthetic incorporation of PBN within mesoporous silica (PBN@SiO_2_) was also studied; the morphology of these particles was characterized using TEM, SEM, XRD, and XPS. The as-made PBN were studied as a fluorescent quencher. The quenching ability of the materials is found to be a function of arsenic(III) concentration; this result suggested a novel application of PBN for fluorescence sensing of arsenic. In addition, XPS studies confirmed that arsenic is adsorbed on PBN@SiO_2_ as arsenite (As(III)) and arsenate (As(V)) irrespective of the initial oxidation state of the material; this result indicated a novel application of PBN for the removal of arsenic(III) from a given sample.

## Figures and Tables

**Table 1:** Parameters calculated from BET nitrogen gas adsorption isotherm.

Sample Name	Surface Area ×10^4^ cm^2^/g	Pore Size (nm)
SiO_2_ Bead	474.8	6.1
PB@SiO_2_ Bead	426.8	4.3

**Table 2:** All arsenic species (As(III), DMA, AsB) identified at different retention times along with their relative concentration during HPLC.

Species	Description	Molecular Formula	Height (%)	Area (%)	System pH	Retention Time	Figure
As^III^	Sodium arsenite	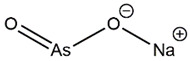	47.1	57.03	2.2	3.86 min	Figure 10B
	(NaAsO_2_)		23.86	30.65	6.8	3.97 min	Figure 10C
			6.91	5.23	8.5	3.95 min	Figure 10D
AsB	Arsenobetaine	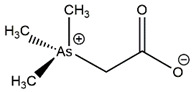	45.56	34.39	2.2	2.55 min	Figure 10B
	(AsB)		58.29	49.74	6.8	2.17 min	Figure 10C
			70.02	56.95	8.5	2.42 min	Figure 10D
DMA	Dimethylarsinic acid (DMA)	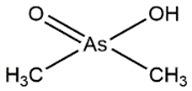	6.85	4.21	2.2	3.58 min	Figure 10B
			15.81	16.21	6.8	3.57 min	Figure 10C
			13.45	11.79	8.5	3.57 min	Figure 10D

**Table 3:** XPS data of PBN@SiO_2_ and Arsenic-PBN@SiO_2_.

Sample	Si(2p)	O	Fe^+3^	Fe^+2^	Fe^+2^	N	As(III)	As(V)
		1s	2p_3/2_	2p_1/2_	2p_3/2_	1s	3d	3d
**PBN@SiO_2_**	103.63	532.62	712.12	721.27	708.34	397.07	-	-
**As-PBN@SiO_2_**	103.49	531.99	712.86	721.42	708.19	397.05 and 398.99	43.23	49.03

**Table 4:** Table with HRSEM, XRD and FTIR data displaying the variations between unused and recycled PBN@SiO_2_.

Analysis	Property	Unused PBN@SiO_2_	Recycled PBN@SiO_2_
HRSEM	Shape	Nanocubic (82%)	Nanocubic (19%)
		and Spherical (18%)	and Spherical (81%)
HRSEM	Size	70–20 nm	17–26 nm
XRD	2-Theta (Planes)	17.6 (200), 24.3 (220), 37.83 (400)	17.4 (200), 24.6 (220), 35.12 (400)
FTIR	CN Str.	2096 cm^−1^	2054 cm^−1^

## Data Availability

Data supporting reported results can be found in the laboratory of Prof. Prem C Pandey of IIT(BHU). https://iitbhu.ac.in/dept/apc/people/faculty.
